# Isolated diffuse hyperplastic gastric polyposis presenting with severe anemia

**DOI:** 10.1186/1757-1626-1-130

**Published:** 2008-08-28

**Authors:** Suriya Jayawardena, Dharshan Anandacoomaraswamy, Olga Burzyantseva, Muhammad Abdullah

**Affiliations:** 1 Coney Island Hospital, 2601, Ocean Parkway, Brooklyn, NY 11235, USA

## Abstract

**Introduction:**

Gastric polyps exist in a wide variety of types, most of which are small and often benign. Discovery of gastric polyps during Endoscopy necessitates biopsies.

**Case presentation:**

We present a case report of an isolated diffuse hyperplastic gastric polyposis in a 26 years old Hispanic female when she was investigated for profound anemia. The Esophagogastroduodenoscopy revealed numerous gastric polyps filling the entire stomach. She was treated with near-total gastrectomy and her anemia resolved

**Conclusion:**

Isolated diffuse hyperplasic gastric polyposis with normal gastrin level is a rare entity and can present with severe anemia.

## Introduction

Gastric polyps include hyperplastic polyps, adenomatous polyps, and inflammatory polyps unlike polyps of the colon are rare and have an incidence of less than 1%. Endoscopic excision of gastric polyps provides a minimally invasive approach to diagnosis and treatment. Hyperplastic polyps are the most common histologic type found among gastric polyps. The association of hyperplastic polyps and anemia has not been well established.

## Case Report

A 26 years old Hispanic female with no significant past medical history came to the emergency room with complaint of weakness and lethargy for 3 weeks. She denied nausea, vomiting, abdominal pain, melena, rectal bleed, heavy menstrual periods or loss of weight. Family history was significant for father having colon cancer at the age of 50 yrs.

**Figure 1:**
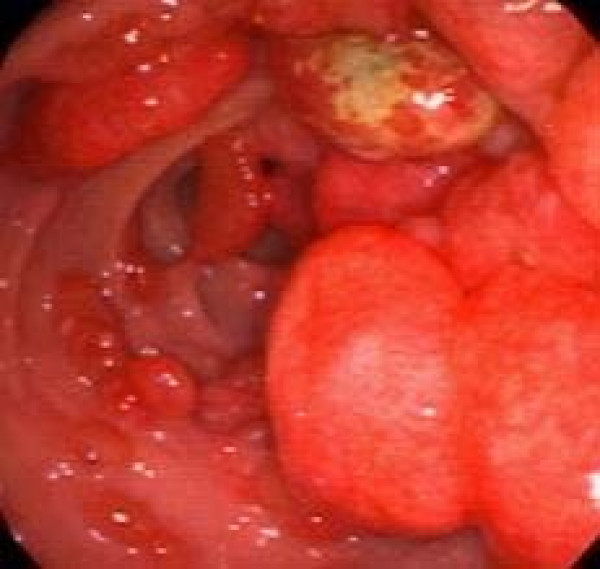
**The Esophagogastroduodenoscopy revealing numerous diffuse polyps of varying size and shape filling the stomach.** Some of the polyps actively bleeding.

**Figure 2:**
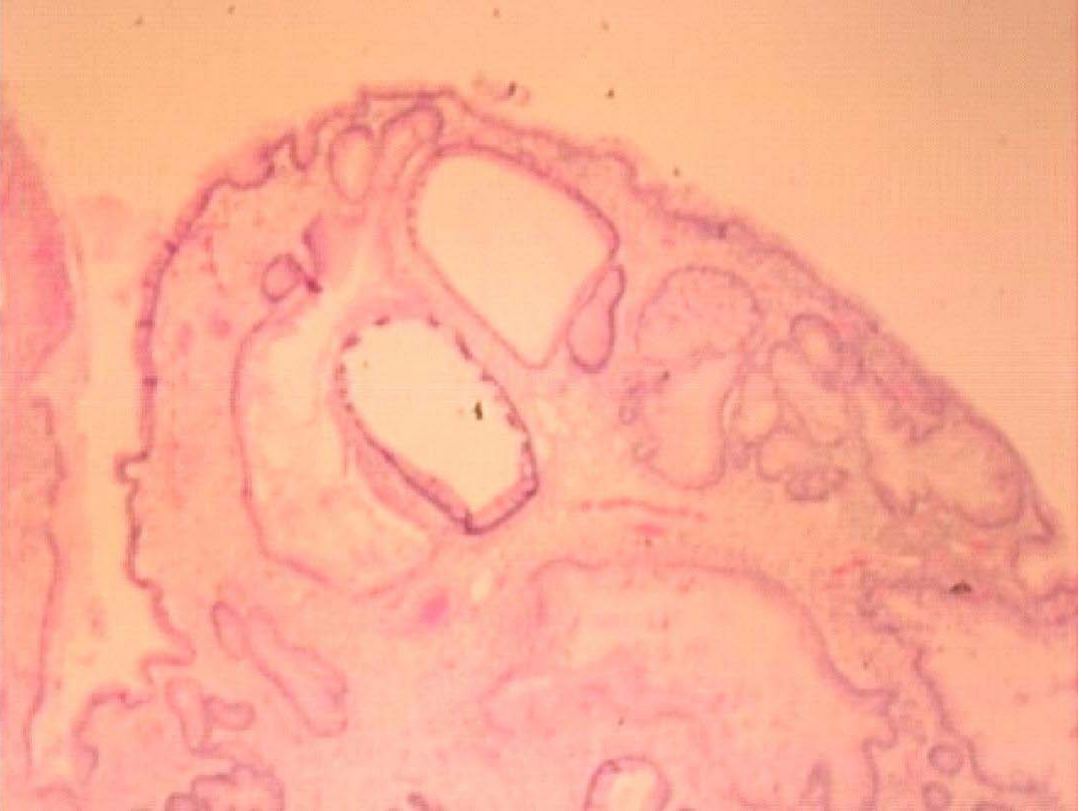
The Histology of the polyps showing dilated, complex tortuous gastric foveolar type glands and intestinal metaplasia with surface chronic ulceration and inflammation.

**Figure 3:**
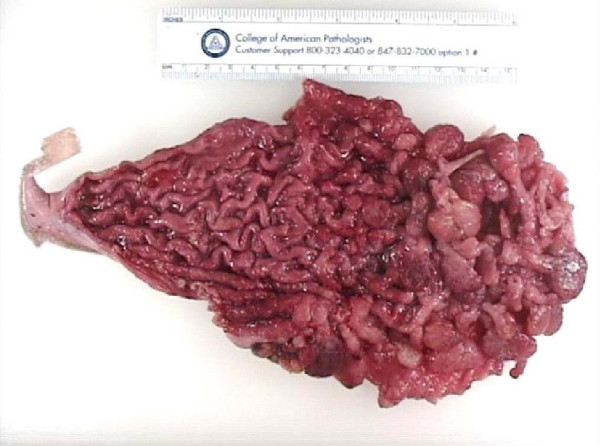
The specimen of the stomach with multiple polyps.

Examination revealed marked pallor, koilonychia and a 3/6 systolic flow murmur at the left sternal border. Her stool occult blood test was positive with brown stools. Laboratory data revealed severe iron deficiency anemia with the Hemoglobin of 4.5 g/dl, Mean corpuscular volume of 50.3 fL. The RDW was 29.8 and the reticulocyte count of 3%. On further investigation the serum Iron was low at 10 mcg/dl, Total Iron Binding Capacity (TIBC) was elevated at 441 mcg/dl and the transferritin saturation was low at 2.3%. Serum ferritin was 3.9, the Vitamin B 12 level was 1469 pg/ml and all other biochemical work up for Anemia was normal. As part of Iron deficiency work up endoscopy was performed. The Esophagogastroduodenoscopy revealed numerous diffuse polyps of varying size and shape filling the stomach. Some of the polyps were actively bleeding. (Fig 1). No polyps were found in the duodenum or proximal jejunum. The serum gastrin level which was done subsequently was normal (47 pg/ml). Serological testing for *Helicobacoter Pylori *was negative. The biopsy of the polyps showed dilated, complex tortuous gastric foveolar type glands and intestinal metaplasia with surface chronic ulceration and inflammation (fig 2) The diagnosis of hyperplastic gastric polyposis was made and the microscopy was negative for *Helicobacoter Pylori*. The Upper GI series and colonoscopy did not show any polyps in the small bowel or colon respectively. In view of the active bleeding polyps and symptomatic anemia patient underwent a laparoscopic near-total gastrectomy with Roux en Y gastric bypass surgery after multiple blood transfusions (fig 3). After the procedure the patient's anemia improved, there was no drop in her hemoglobin and hematocrit in the subsequent follow up in the clinic for the past two years.

## Discussion

Gastric polyps are rare as compared to colonic polyps (Scott-Conner 2006) [1]. Among the different types of Gastric polyps the hyperplastic polyps are the most common. Although these hyperplastic polyps do not harbor malignancy, there is rare chance of malignancy especially when associated with pernicious anemia (Rickes, Gerl et al. 2000) [2]. Gastric polyps can also be part of polyposis syndromes such as juvenile polyposis, Gardner, Peutz-Jeghers, and Cronkhite-Canada syndromes. Diffused gastric polyposis is a rare entity with only a few cases being reported (Hu, Hsu et al. 2002) [3]. These polyps also run in families and are a part of familial polyposis syndromes. In our patient, she herself did not have a history of gastrointestinal adenoma or carcinoma but the father had colon cancer. The other major association of gastric polyps is with pernicious anemia (Rickes, Gerl et al. 2000) [2]. Thermal injury to the stomach seen in laser therapy for watermelon stomach can also give rise to gastric polyps (Geller, Gostout et al. 1996) [4]. Gastric polyposis can present with iron deficiency anemia like in our patient, hypoproteinemia, high gastrin levels and even gastric outlet obstruction (Kepczyk and Kadakia 1995) (Covotta, Paoletti et al. 1995) [5,6]. Gastritis associated with Helicobacter pylori infection can also lead to gastric polyps which are amenable to H. pylori eradication therapy (Isomoto, Furusu et al. 2005) [7]. Use of proton pump inhibitors, especially in children can give rise to gastric polyps and/or nodules (Pashankar and Israel 2002) [8].

Association between Hyperplastic gastric polyposis, hypergastrinemia and colorectal malignancy has been described, but in our patient the serum gastrin level was normal (Niv, Delpre et al. 2003) [9]. Another interesting observation is the development of hyperplastic gastric polyps in patients who undergo solid organ transplantation and immunosuppressive therapy. The association of these polyps with the immunosuppressive therapy has not yet been well established (Amaro, Neff et al. 2002) [10]. Our patient was not exposed to any immunosuppressive therapy and did not undergo any organ transplant.

## Conclusion

Isolated diffuse hyperplasic gastric polyposis with normal gastrin level is a rare entity and can present with severe anemia. Total gastrectomy or near-total gastrectomy to prevent further occult blood loss and regular surveillance with endoscopy is necessary as there is a possibility of malignancy developing in these polyps.

## Abbreviations

GI: Gastro Intestinal; RDW: Red Cell Distribution Width; TIBC: Total Iron Binding Capacity.

## Competing interests

The above case report was written at Coney Island Hospital. The above mentioned authors have no affiliation to any other institute other than Coney Island Hospital.

## Authors' contributions

SJ, DA and OB treated the patent and were responsible for writing the paper and looking up the back ground references. MA was responsible for over all coordination and final proof reading. All the above mentioned authors read and approved the final manuscript.

## Consent

A written informed consent was obtained from the patient for publication of this case report and accompanying images. A copy of the written consent will be made available on request
